# Dietary Behaviours of University Students during the COVID-19 Pandemic. A Comparative Analysis of Nursing and Engineering Students

**DOI:** 10.3390/foods11121715

**Published:** 2022-06-11

**Authors:** Miriam Araujo-Hernández, E. Begoña García-Navarro, María José Cáceres-Titos

**Affiliations:** 1 Department of Nursing, University of Huelva, 21007 Huelva, Spain; miriam.araujo@denf.uhu.es (M.A.-H.); mariajosecacerestitos@gmail.com (M.J.C.-T.); 2 ESEIS Research Group, COIDESO Research Centre, University of Huelva, 2100 Huelva, Spain

**Keywords:** nutrition, university students, dietary habits, COVID-19

## Abstract

The university stage is a crucial stage that influences the decision-making process of students. At this stage, they acquire dietary habits that are guided by their likes, beauty ideals, biological influences, and economic factors. During the COVID-19 pandemic, universities closed and turned to online teaching, changing their life habits under the duress of confinement. The aim of the present work was to identify the dietary behaviours of nursing and engineering degree students at the University of Huelva during the period of confinement, in addition to identifying the factors influencing these habits. The methodological strategy employed was mixed in nature. In this sense, a cross-sectional descriptive study was first performed, followed by a phenomenological qualitative study that was descriptive in nature. Examination of outcomes revealed the presence of four lines of argument founded on the influence of context, life habits, emotional changes experienced during the COVID-19 pandemic, and the factors facilitating or limiting adaption to this period. Taking into account that confinement, restrictive measures, the absence of family, closeness and affection, and training influenced changes to feeding habits and approaches to consumption, universities could carry out interventions oriented in this line to favour healthy eating habits.

## 1. Introduction

In the same way as the rest of the world, Spain experienced substantial changes at all levels due to the widespread infection caused by the outbreak and almost generalised spread of the SARS-CoV-2 coronavirus [1]. In January 2020, the World Health Organization (WHO) declared the spike of disease caused by the new coronavirus (COVID-19) to be an internationally relevant public health emergency. In March 2020, further evaluation carried out by WHO deemed COVID-19 to be a pandemic [2]. Following a declaration of a state of emergency on the 14th of March, the Spanish government enforced mandatory confinement on the entire Spanish population, with the exception of individuals employed in professional activities classed as being essential, such as health professionals, security and defence forces, health sector professionals, and those working in other specified activities. In general, confinement could only be broken to purchase food, cleaning and hygiene products or other bare necessities, take out the rubbish, or walk the dog. All remaining time had to be spent within the home address [3].

Universities, along with other educational institutions, were forced to close their doors and turn to online working for both teachers and students. This led them to change their overall approach under duress by the situation enforced by confinement.

The population of university students, in itself, is made up of highly vulnerable groups due to physiological processes inherent to their generational age, alongside the sociocultural changes resulting from moving home, independence, and a changing economy [4]. The university stage has a determining influence on the decision-making of students. It defines a point at which young people autonomously acquire dietary habits, mainly guided by their personal likes, biological influences, and peer group, whilst also being related to economic factors and the desire for a healthy body, amongst other aspects [5,6]). Different studies [7,8] agree that student dietary habits are characterised by a large consumption of foods with a high-density energy content (fizzy and sugary drinks, fried foods, ultra-processed and fast foods, sweets) and an excessive consumption of meat and its derivatives. Such foodstuffs are favoured over fresh foodstuffs and those subjected to a low level of industrial processing, with a low consumption of legumes, fish, fruit, and vegetables. Other studies [9,10,11] pertaining to the health sciences university population have concluded that nutritional knowledge instilled through academic courses do not translate to healthy lifestyles, with high calorie-content and nutritionally imbalanced foods being consumed [9,10,11].

During imposed confinement, the Spanish Society of Community Nutrition (SENC) laid out nutritional and physical activity recommendations as a means of helping individuals adapt their nutritional and physical activity behaviours to the new situation. The avoidance of impulse buying, comfort eating and overeating of ultra-processed foods was one of the main objectives of these guidelines. Another objective was to promote different types of physical activity to be performed at home or on terraces and consider sources of vitamin D and specific foods of interest for the immune system [12].

With regards to the mobility restrictions imposed by having to stay at home, various studies [13,14] state that reduced mobility directly influences the immune system and mood state. Specifically, psychological behaviours associated with the virus and its origin are described, including food hoarding and the stigmatisation of infected individuals [15]

Other studies [16,17] argue that moderate intensity exercise is associated with a reduced incidence, duration, and severity of upper respiratory tract infections. Although these outcomes have not been examined in individuals infected with SARS-CoV-2, it can be inferred that similar patterns would emerge.

Another consequence resulting from confinement of the population and, therefore, of the university population, includes alterations to the mood state [18]. Specifically, it is possible that confinement led to an increase in difference dysfunctional feeding patterns, such as the consumption of food as a means of distraction or a masking strategy to cope with negative emotions [19,20]. Some studies [21,22] have examined the effects of confinement on university students attending different institutions. In Spain, the University of Granada [21] made use of the outcomes of the aforementioned study to design health promotion strategies targeting nutrition following confinement. Another study developed by the Open University of Catalunya and the University of Valencia [22] produced interesting information by comparing the nutritional habits of students studying health sciences and those studying other sciences at the same campus. This study identified a direct relationship between health knowledge and better health habits, greater physical activity engagement, and better Mediterranean diet adherence in students undertaking health sciences.

Within the proposed context, the aim of the present work was to describe the nutritional behaviours of students undertaking nursing or engineering degrees at the University of Huelva during the period of confinement, whilst also identifying the factors that condition these habits.

## 2. Materials and Methods

The methodological strategy employed was mixed in nature, first, employing a descriptive cross-sectional study with students undertaking the last year of nursing or computer science and engineering courses. Following this, a phenomenological qualitative study was conducted via a similar content analysis to that described by Taylor and Bodgan [23].

### 2.1. Study Population

With regards to the quantitative approach, a survey study was conducted using a representative sample in terms of general data, with a maximum sampling error of 5.35%. Given the high representation of women in the population of students undertaking nursing degrees and the high representation of men undertaking computer science and engineering degrees, it was intended to apply proportional gender allocation methods at each university, obtaining a final sample of 139 individuals, of which 90 were women and 49 were men, and of which 85 were nursing students and 54 were engineering students. However, given the exceptional circumstances imposed by confinement during the period of data collection, an ad hoc questionnaire had to be used via Google Forms during the months of April and May 2020.

As can be observed in Table 1, the exceptional state of events meant that the gender allocation ultimately achieved did not exactly correspond to the distribution found within the population. Other similar studies have reported experiencing similar issues [24,25].

With regards to the qualitative approach, intentional sampling was performed via a snowballing approach carried out within the overall population, with data being stratified according to university and gender. The sample size was determined progressively over the course of the research study, with the final sample being finalised once saturation of new information was achieved.

Inclusion criteria stipulated that participating students were undertaking the final year of a nursing or computer science and engineering degree course, had an active registration during the pandemic, and provided informed consent to voluntarily participate in the research. Sociodemographic variables were considered for all participants.

The study followed the international ethical recommendations laid out in the Declaration of Helsinki. Participation in the study was completely free and voluntary. Responses to all questionnaires were anonymous, and all provided personal information (name, ID) was stored in a manner that complied with legal requisites for the protection of personal data and guarantee of digital rights (Organic Law 15/1999, 13 December 1999 and Organic Law 3/2018, 5 December 2018). The project was approved by the Ethics Committee of the Council of Andalusia (reference number: 183-N/2020 PEIBA).

### 2.2. Data Collection

For the preliminary study, the survey administered comprised a validated nutritional behaviour scale [26] that was specifically targeted towards the university population. The scale collected data on feeding times, consumption preferences regarding foods and drinks, the way in which foodstuffs were prepared, the reading of nutritional labels, food consumption outside of the home, satiety, the following of therapeutic or special diets, and perceptions of healthy nutrition, barriers to change, and beliefs. The questionnaire was completed online via Google forms©. This enabled it to be disseminated through WhatsApp© via the use of different strategies by course leaders. It was also disseminated through the Moodle teaching platform, with these approaches being key to the delivery of the study during the period of confinement.

For the qualitative study, given the exceptional set of circumstances present during the performance of field work, it was impossible for researchers to approach informants in person. As a result, interviews were performed through remote video and audio connection via the program Zoom©, with sessions being recorded from start to finish from the moment at which participants provided consent to participate in the interview. Three research team members participated in the delivery of interviews. The aim of this was to minimise the implicit risk of the absence of physical contact between actors, which can lead to a failure to perceive non-verbal expressions, with resultant effects on analysis. One researcher proceeded to conduct the interview whilst the other two took charge of the technical aspects and took field notes. A total of 6 in-depth interviews and 2 focus groups were conducted. The research team moderated group discussion and interview development, whose progress was based on a number of scripted questions. Interviews lasted approximately 40 min. This analysis enabled the aspects most relevant to elaboration of the interview guide and the question at the heart of focus groups to be examined in greater depth.

### 2.3. Data Analysis

Survey data was analysed using the software program SPSS version 20 belonging to IBM©. Results are presented in the form of means, standard deviations and distributions. Differences between sub-groups were evaluated using chi-squared analysis for discrete variables. When performing this analysis, a weighting factor was applied, which enabled more proportional representation of examined strata relative to the distribution found in the actual population. This factor was developed in consideration of the distribution seen in the target population according to sex and degree undertaken.

With regards to discourse analysis, following the literal transcription of interviews and discussion groups, further analysis was conducted that was based on the model outlined by Taylor-Bodgan [26]. In this phase, a deductive coding process was followed based on the obtained information and its classification according to dimensions, categories, and sub-categories. At the same time, participants were assigned an alphanumeric reference number, which served for the recording of data and the elaboration of the aforementioned categories and sub-categories. These provided the base unit of analysis composed of various lines or phrases that exposed a central idea extracted from the interviews. Preliminary categorisations were performed manually using the computer program Atlas.ti. 8.0.

Following this, data was triangulated. The different techniques employed provided obtained data with greater validity and reliability.

## 3. Results

The analysed sample was comprised of 139 individuals, of which 90 were women (64.7%) and 49 (35.3%) were men. Obtained outcomes pertained to 85 nursing students and 54 engineering students. The age of interview informants ranged from 21 to 39 years old, with 95% of individuals being younger than 30 years old and 82% being younger than 25 years old. Average age was 23.73 years, whilst the mode was 22 years.

Of the surveyed students studying nursing, 72 were women (84.7%) and 13 were men (15.3%). Of those who were studying engineering, surveys were completed by 18 women (33.3%) and 36 men (66.7%).

A total of 22 informants were interviewed (Table 1). Informants were distributed between six in-depth interviews and two group interviews (discussion groups), with the latter being composed of eight students each from both examined disciplines (nursing and engineering). The sample was heterogenous and representative of the examined population.

Principal component analysis (PCA) revealed the existence of four main lines of argument on which informant discourse was hinged. These lines of argument addressed the aims of the present study and pertained to context, lifestyle habits, emotional changes experienced during the COVID-19 pandemic, and the factors that facilitate or limit the ability to adapt to this period of time (Table 2).

The first line of argument refers to the personal, family, and environmental context in which the COVID-19 pandemic was lived out, alongside the nutritional decisions and approaches to feeding that characterised this time in light of this context.

When analysing the family setting, the accompaniment received by students during the COVID-19 pandemic was described with regards to whether they lived with family, alone, or with flatmates, in addition to which of these normally prepared meals.

A total of 66.2% lived with their parents and 24.5% lived with flatmates. Of those living with their parents, 41.7% pertained to nursing degree students and 24.5% to engineering students. Of the 66.2% of those who were interviewed who were living with their parents, 53.2% indicated that either their mother or father cooked the food they ate. Likewise, of those individuals who responded that they did not live with their parents, 25.2% indicated that they cooked their meals themselves.

When addressing feeding behaviours, the types of foodstuffs consumed and their preparation (fried, steamed, or boiled, roasted or grilled, baked, stewed, or sautéed), responses revealed that more than half of students (55.4%) preferred roasted or grilled food, relative to 23.7% who preferred stewed or sautéed. When comparing the responses given by students on the two types of degree course, the option “fried” was selected by 14.8% of engineering students relative to only 2.4% of nursing students who opted for this response. These differences were found to be statistically significant (*p* < 0.05).

Further, the avoided foods and the reasons behind this were also examined. A total of 74.6% of nursing students avoided some type of food in an effort to take care of themselves, whilst 63.6% of engineering students responded that they did not tend to avoid any type of food.

When this topic is considered from the phenomenological perspective, more in-depth information is uncovered about the impact of the COVID-19 pandemic on feeding habits. Discourse analysis on the influence of isolation on food consumption was manifested through a total of 34 codes. The personal context (16) describes the way in which personal beliefs influence the value placed on feeding in daily life. The family setting (12) describes the accompaniment perceived by students during the COVID-19 pandemic (family, alone, or with flatmates) and the changes caused by this accompaniment with regards to their dietary habits. The environmental context (6) described the physical place and circumstances surrounding individuals during confinement. Analysis focused on whether or not a physical separation existed between the settings related with feeding and study, in addition to whether these settings were influenced over time (Table 3).

The second line of argument refers to the lifestyle habits followed by students prior to the COVID-19 pandemic and the way in which they changed or were modified during confinement.

Addressing habits related with body image and caring for one’s body, differences found between the two examined student groups stood out. A total of 56.6% of nursing students employed strategies pertaining to taking care of nutrition, exercise, or the acquisition of a healthy diet, relative to 32.4% of engineering students who employed these strategies. This reveals notable gender differences, with 69.7% of women providing this response.

When this line of argument is considered from a phenomenological perspective, three codes emerge to describe informant discourse, namely, the physical exercise engaged in (6), the online classes (18) that had to be attended during this time, and the use of social networks and apps (5).

With regards to physical activity, it serves to highlight some of the modifications resulting from the pandemic with regards to the location chosen for this activity and the intensity of activity. Two contrasting elements were observed. The first pertained to individuals who engaged in exercise prior to the pandemic and were faced with challenges to continuing with the same routine during confinement. The second pertains to references to sedentary behaviour and the influence of online teaching on the failure to achieve these prior routines.

Although some informants referred to being able to continue with their exercise routines via the internet or exercise apps, all informants agreed that differences inherent to this new modality with regards to its effectiveness were related with the challenge of performing exercise at home, lack of resources, etc. Further, rising sedentary behaviour due to learning at home led to an increase in the calorie content of the diet.

Another worthwhile dimension to highlight is related with the use and abuse of social networks during confinement. All interviewed informants mentioned that this behaviour had affected their dietary habits, sometimes due to adopting a new routine, in other cases due to anxiety and, sometimes, even due to the challenge itself of following some of the publications they saw on social networks. In fact, the discourse mentioned that the profiles of some influencers dedicated to nutrition had seen in a rise in the number of followers from this target university group (Table 4).

The third line of argument described the emotional changes suffered during this period. Consideration is focused on the three codes proposed to condition these changes, namely, the confinement (10) experienced during the first three months following the decreed state of emergency, uncertainty generated by the pandemic (9), and the social distancing enforced by the health crisis (7) (Table 5).

One of the questions posed was focused on the emotional reaction experienced when feelings of satiety were achieved following food intake. A total of 63.5% of nursing students mentioned that they were able to stop eating when they were full, relative to 11.7% who reported the need to continue eating even after feeling satisfied. When the same question was posed to engineering students, 38.9% reported that they would continue eating despite feeling satisfied.

Questions were also asked with regards to the types of foods consumed when intake occurred between meals as these behaviours are associated with feelings of stress and anxiety. Obtained outcomes pointed to a significant difference between the two examined student groups (*p* = 0.038). Nursing degree students preferred to snack on fruit (30.6%), yoghurt (10.6%), and nuts (32.9%), compared with engineering students who preferred to consume sweets (18.5%) and nuts (18.5%) outside of meal times.

Considering this finding from a phenomenological perspective, discourse stands out that was generated around emotions and the consumption of certain foods, with this being shown to increase at certain times during confinement. In some interviews, informants described periods of particular vulnerability when faced with the uncertainty of what was going on and the ongoing changes taking place around them (university teaching, family situation, etc).

Some recorded discourse gave voice to the negative thoughts that were taking over positive thoughts and starting to play a leading role as the pandemic progressed. Both the mass media and social networks were reported to have directly influenced mood states and, at the same time, dietary habits, not only in terms of quality but, also, the quantity of consumption. According to informant discourse, emotional changes directly led to an increase in foods high in sugar and fat.

Confinement, restrictive measures, and absence of the family, closeness, and affect, led to changes in the mood state of those interviewed who also mentioned that their habits, lifestyle, and food intake was related to these factors, alongside their opportunities for socialisation (Table 5).

The fourth and final line of argument reflects elements that were identified as facilitating (8) or limiting (9) dietary consumption during the COVID-19 pandemic.

With regards to this line of argument, students were asked about the factors they considered to be relevant to their consumption with regards to the type of foods consumed, in addition to the factors that conditioned the following of a healthy diet. Outcomes referred to the way in which academic training had exerted a meaningful influence on the motivation to engage in health consumption habits. A total of 35.3% of nursing students reported that nutritional content was the main conditioning factor pertaining to consumption, relative to just 10.1% of engineering students. Nonetheless, 38.7% of these students attributed more value to characteristics related with the taste of food.

Another of the issues addressed by the survey pertained to the elements required by students in their day-to-day lives to equip them to be able to improve their diet. Similar results were obtained between both disciplines (nursing and engineering), revealing values such as commitment and motivation (23.0–15.1%), more information and training (6.5–6.5%), and time (7.2–4.3%), followed by money (4.3–2.9%) and social support (4.3–1.4%).

It serves to highlight that this line of argument demonstrated greater variability in the analysis conducted according to student group than those discussed earlier.

Training was one of the elements identified as being a facilitator, especially by nursing students, who highlighted that training was fundamental to decision-making at the time of making purchases, consuming foods, and consuming snacks outside of meal times. Engineering students contributed an additional important element to that presented up until now, in that they related the personal characteristics, resulting from family feeding habits, of individual students to this aspect. Some of those surveyed highlighted that the profession of their parents or the individuals with whom they shared the period of confinement had influenced their consumption and feeding habits.

With regards to the limiting elements most highlighted by engineering students, obstacles included the lack of training, impact of information received through mass media, advertising, and social networks, behaviour prior to the pandemic, and the internal pressure felt to maintain an expected body image.

Nursing students discussed the stress caused by this situation, alongside the previously defined characteristics (online teaching, changes to the academic calendar and the way in which modules are evaluated, the new location of platforms, platform use, etc.), as the main limiting element over their ability to adapt their dietary habits during confinement (Table 6).

## 4. Discussion

The aim of the present research was to identify the dietary behaviours of students during the period of confinement, whilst also conducting a comparative analysis of students undertaking nursing and engineering degrees at the University of Huelva. The mixed approach to this issue enabled not only student habits to be uncovered, but also permitted the reasons behind them and the intentions of them to be revealed.

The present study identified four lines of argument that describe student behaviour with regards to nutrition. The first of these was related to the individuals with whom students lived, specifically the family or flatmates, with the family context proving to be more protective against unhealthy nutrition given that parents tended to be responsible for preparing meals. This coincides with the outcomes of other research studies [27,28] conducted during the same period of confinement in other international contexts. Both of these previous studies concluded that family support favoured engagement in healthy nutrition.

With regards to the lifestyle, the present study closely aligns with other previous research [29,30,31,32] in its description of the way in which the dietary habits of Spanish adults demonstrated some healthier traits during confinement due to COVID-19. Positive change was characterised by increased consumption of vegetables, fruit, legumes, and fish, in contrast to other groups, which demonstrated increased tendencies towards a greater consumption of alcoholic or sugary drinks and processed foods containing high amounts of fats, salt, and sugar. Groups adopting such tendencies also experienced negative changes to their physical activity habits. In the present population, this pattern emerged to a greater extent in students with less training on healthy habits and those undertaking an engineering degree.

On the other hand, when habits related with body image and taking care of one’s body, differences were found in the present study in both student groups, although nursing degree students were more concerned about taking care of themselves in this sense. A study conducted by the University of Edinburgh during confinement [33] described the perceived impact of the shutdown caused by COVID-19 in the United Kingdom with regards to diet, exercise, and body image. The study revealed that women and, above all, younger women, were disproportionately more likely to report changes to their thoughts and behaviours. Such changes included the greater challenge of controlling their diet, being more concerned about food, and being increasingly concerned about their appearance. This finding can be extrapolated to the present sample in the sense that, in the present population, this same outcome emerged within nursing students who were mostly women. Other similar studies [34,35] explain these outcomes by the fact that women tend to assume more responsibility over care provision, leaving them more exposed to the stigmas related with weight spread through public health messages and social networks.

The third line of argument to emerge from the present research pertains to the emotional component of nutrition in university students during confinement. Similar studies [36,37] have associated the lack of social interaction with peers, teachers, relatives, and friends with emotional instability in students. This factor emerged in the present research, which also proposed the novel factor of the “digitalisation of university teaching” as a catalyst of the aforementioned factor, potentially leading to an increase in stress, anxiety, and sleep disorders, alongside a reduction in self-esteem in students. This, at the same time, leads to the acquisition of unhealthy lifestyle habits such as increased energy intake and reduced physical activity engagement.

A study carried out at a leading public university in the United States during the pandemic [38] evaluated the severity of anxiety symptoms and its association with dietary traits in students. Of the 1243 students to complete the survey, 51.9% reported moderate to serious symptoms of anxiety. Most serious anxiety symptoms were associated with increased hunger, emotional overeating, a reduced response to satiety, and diminished enjoyment of food. This phenomenon was also seen in the present study, with anxiety resulting from confinement being dealt with through disordered eating habits and continuing to eat regardless of satiety. This was born out through data showing that, while 63.5% of nursing students reported being able to stop eating, 11.7% stated a need to continue eating after feeling full.

The final line of argument to emerge from the present study pertains to factors considered by the student to be relevant to their consumption with regards to types of food and the individuals who motivate them to follow a healthy diet. It is notable that this line of argument demonstrated greater variability than the others when examined according to student group. In this sense, nursing students viewed academic training as a crucial building block on which to make decisions, form judgements about the nutritional content of foodstuffs, and understand the pathologies derived from poor nutrition. In stark contrast to this, engineering students reported that the overriding factor driving their consumption of a certain food type over another was the taste of the food. A study conducted at universities in Ecuador and Peru [39] also reported a foundational effect of academic training on the acquisition of healthy habits in that it led students to assume responsibility over taking care of themselves.

## 5. Conclusions

The health status of the university population is built on the synergistic relationship between healthy lifestyle habits and the personal and material resources on hand at any given time. Present findings describe the way in which confinement influenced the dietary habits of university students and the role played in relation to these changes by the acquisition of knowledge of healthy habits. In this sense, nursing degree students were more likely to continue to follow healthy dietary habits than engineering students.

Confinement, restrictive measures, and the absence of family and opportunities to experience closeness and affect led to changes in the mood state of those interviewed who, in turn, stated that their habits, lifestyle, and dietary intake were related both with these aforementioned factors and with their opportunities for socialisation. Future lines of research should be aimed at deepening factors such as loneliness and recreational deficits that influence feeding habits.

Elements considered to be promotive of healthy habits were deemed to be influenced by student training with regards to the diet and lifestyle. Training was highlighted to provide students with greater confidence at the time of making decisions when making food purchases and choosing the types of foods to eat, in addition to impacting decisions around food consumption outside of regular meal times. Students with less knowledge about nutrition pointed to the personal characteristics of individuals as the main catalyst, with these being born out in perceived family dietary practices. Universities must include training actions not only in food, but in healthy shopping and empowerment and self-esteem to help in decision-making.

Based on the findings of the present study, the acquisition of cognitive, attitudinal, and experiential skills by university students with regards to healthy habits will enable the development of strategies targeting the adoption and maintenance of healthy habits in the university community.

### Limitations

A limitation of this research implies the ability to generalize the results obtained to other universities and even to other disciplines not included in the study, although it has allowed us to establish actions that can be extrapolated to other university communities.

## Figures and Tables

**Table 1:** Sociodemographic characteristics of participants.

Participant	Degree	Age	Sex	Online Teaching	Training in Nutrition	Participation
Participant 1	Nursing	25	M	X	X	Interview
Participant 2	Nursing	20	M	X	X	Interview
Participant 3	Nursing	21	F	X	X	Interview
Participant 4	Comp Scien Engineering	22	M	X		Interview
Participant 5	Comp Scien Engineering	22	M	X		Interview
Participant 6	Comp Scien Engineering	22	F	X		Interview
Participant 7	Nursing	21	M	X	X	Focus Group 1
Participant 8	Nursing	22	M	X	X	Focus Group 1
Participant 9	Nursing	21	F	X	X	Focus Group 1
Participant 10	Comp Scien Engineering	23	M	X		Focus Group 1
Participant 11	Nursing	23	F	X	X	Focus Group 1
Participant 12	Comp Scien Engineering	22	F	X		Focus Group 1
Participant 13	Comp Scien Engineering	21	M	X		Focus Group 1
Participant 14	Comp Scien Engineering	23	F	X		Focus Group 1
Participant 15	Nursing	21	M	X	X	Focus Group 2
Participant 16	Nursing	22	M	X	X	Focus Group 2
Participant 17	Comp Scien Engineering	23	M	X		Focus Group 2
Participant 18	Comp Scien Engineering	21	M	X		Focus Group 2
Participant 19	Nursing	21	F	X	X	Focus Group 2
Participant 20	Nursing	22	F	X	X	Focus Group 2
Participant 21	Comp Scien Engineering	23	M	X		Focus Group 2
Participant 22	Comp Scien Engineering	21	F	X		Focus Group 2

M = Male F = Female.

**Table 2:** Description of the categories, codes, and number of citations produced by analysis using Atlas ti.

Dimension	Line of Argument	Code	N. Citations
Dietary habits	Context	Personal	16
		Family	12
		Environmental	6
	Lifestyle habits	Exercise	6
		Online teaching	18
		Social networks	5
	Emotional changes	Confinement	10
		COVID-19 pandemic	9
		Social distancing	7
	Determining factors	Facilitative	8
		Limiting	9

**Table 3:** Codes and nursing and engineering student discourse on the influence of the personal, family, and environmental context.

Line of Argument	Code	Citations	Degree	Discourse
Contexto	Personal	10	Nurse	In my case, for example, it hasn’t affected me, for other people. It’s just that dietary habits are really influenced, above all, by the emotional state and stress. There are a lot of compensate for stress with binges, for example, or the work routine, which influences a lot and it is difficult to change attitudes that are so deeply rooted within individuals.
		6	Computer Science Engineering	Well, for me it is a really big change because before I was in Faro, Portugal and here in Huelva, that was already a really really drastic change.
	Family	9	Nurse	At the beginning the stress was huge because I wasn’t able to coordinate work with my day-to-day and I spent all day inside at home, as well as suffering because my mother was at home and I felt like being with my family, because if my family is working as well as me, then the day passes more quickly.
		3	Computer Science Engineering	During confinement, I was living with my parents, something that I wasn’t doing before. My mum normally cooked and we basically ate soups and stews, anything with a spoon.
	Environmental	2	Nurse	Knowledge, family, the culture in which you find yourself… if your family, above all those who you live with at home, follow a healthy diet, you will too.
		4	Computer Science Engineering	Yes, it’s true that being in front of a screen all day starts to take effect and lowers your mood. As a result, that was an element that made me change.

**Table 4:** Codes and nursing and engineering student discourse on lifestyle habits, with regards to physical exercise, online teaching, and social networks.

Line of Argument	Code	Citations	Degree	Discourse
Lifestyle habits	Exercise	5	Nursing	What has affected me has been, for example, moving from engaging in sport in a gym to doing it at home. That has really affected me a lot. Not being able to walk in the street, which really is an important physical activity. On a normal day it it really possible that we can walk almost 100 thousand steps. That has affected me in that my energy expenditure is less, it’s not the same training at a sports centre as at home, well I’ve had to cut down and reduce my food energy intake, because when I was still with the same energy intake and lower expenditure, my weight was increasing.
		1	Computer Science Engineering	Confinement has caused me to move less. Before I was walking to university and now that I don’t have to go to class, well, I move less. Due to not moving a lot, I noticed when I went out to walk, I noticed that my legs were stiffer, my back hurt.
	Online teaching	9	Nursing	Whether I have to attend and online class, whether I don’t have to attend, what work I have to do. Maybe you were relaxed eating and you got an email from a teacher saying “this work for next week”. Then that would cause anxiety and some brutal emotional and sentimental changes. Teaching, COVID, as I mentioned a bit before, teaching has added an extra challenge to good nutrition. Teaching has affected me quite a lot because it was a huge effort for me to adapt to online teaching and I think that was difficult for everybody, as much for teachers as for us as students, a lot of work has changed a lot, and with online exams being new and having little time to adjust, we didn’t know how to study for them.
		9	Computer Science Engineering	As time went on and exam time became closer. Then I started to eat less as well due to stress more than anything.
	Social networks	2	Nursing	Social networks, during confinement I have used them to a ridiculous and totally unecessary extent. Because on top of that it was, you’d go onto Instagram and you’d see the same thing over and over again, time after time, with WhatsApp, the same conversations, making unecessary calls just because one is bored, it was out of anxiety. I was calling friends and making conversations go on longer just to be in touch, even though it was through a screen with another person.
		3	Computer Science Engineering	I follow other accounts to do with sport, motivational phrases, healthy nutrition, as little recommendations. I know that Carlos Ríos is trained and is backed by a team.

**Table 5:** Codes and nursing and engineering student discourse on the emotional changes experienced during the COVID-19 pandemic.

Line of Argument	Code	Citations	Degree	Discourse
Emotional changes	Confinement	5	Nursing	Confinement has led to quite a lot of change in my life. I have changed my dietary habits, my physical condition with regards to sport… Before confinement, for example, I didn’t do any sport, after confinement, I do. It has been like a pause, to decide what is really important, what isn’t important and you really do realise that we don’t need so many gadgets or superficial thinks in order to live.
		5	Computer Science Engineering	At first, I went a bit off course because I tend to go out at weekends with my friends and not being able to go out and being locked in the house all the time, well, that really was a little bit frustrating.
	COVID-19 pandemic	6	Nursing	Prior to confinement, my emotions were more or less stable, in that I was going out with my friends, with my partner, to university, had a good relationship with my teachers and classmates. However, having all of this elude you and being confined in your own house, it was pretty hard because one day you are missing your partner, you would get sad.
		3	Computer Science Engineering	I kept doing my training as well as I could at home, but well, it’s not the same, you know. I also saw, with regards to my feelings, that it is true that they were quite bad, negative, I was kind of depressed, above all because of the exercise, because before confinement when I was doing sport I felt stronger, more energetic.
	Social distancing	3	Nursing	It’s true that there have been times in which I have gone off track because I am a really social person, so human contact, I truly did miss that a lot.
		4	Computer Science Engineering	At first I went a bit off course because I tend to go out at weekends with my friends and not being able to go out and being locked in the house all the time, well, that really was a little bit frustrating.

**Table 6:** Codes and nursing and engineering student discourse. Facilitating and limiting factors.

Line of Argument	Codes	Citations	Degree	Discourse
Determining factors	Facilitative	3	Nursing	In truth, I have quite a lot more knowledge now than year ago because in nursing, the module “Feeding and Nutrition” has provided me with a lot of knowledge that I never had before, I have been able to dispel a number of myths and it has taught me the basis of healthy nutrition, about what ultra-processed foods are, that I didn’t know before. Then as you continue to grow and you also go on accumulating knowledge, well you also start deciding on the path you should take, whether the nutrition that your family has given you has managed to be healthy or unhealthy, or any other type of diet that you have imposed on yourself, according to the knowledge that you acquire.
		5	Computer Science Engineering	Like my mum who, it bears saying, works as a nurse at the Juan Ramón Jiménez hospital. My mum is knowledgeable and tries to guide my dad and me. She is fantastic, she knows what’s best and she does it.
	Limiting	4	Nursing	I think that stress has an influence.
		5	Computer Science Engineering	If every second advert is selling junk food, for example, well they might just eat more junk food than if they weren’t to see that. During confinement, I moved less, I didn’t sleep as well, my sleep cycle was totally broken. Computers totally condition us. Like, it is a constant between my peers because we are sat down all day. On top of that, although it might seem a cliché, but it sometimes does happen, we tend to gain weight because we are sat down and our work is stressful. Well I have always been a chubby person, I wasn’t fat but I did like it, so I think that your physical appearance can also condition your diet, because having a physical appearance that you like, can encourage you to keep it that way, you eat more healthy. Or if you don’t see yourself as attractive, you encourage yourself to do it.

## Data Availability

Data is contained within the article.

## References

[1] Lu H., Stratton C.W., Tang Y. (2020). Outbreak of pneumonia of unknown etiology in Wuhan, China: The mystery and the miracle. J. Med. Virol..

[2] OMS Organización Mundial de la Salud. Consideraciones Psicosociales y de Salud Mental Durante el Brote de COVID-19. https://apps.who.int/iris/handle/10665/331490.

[3] Ministerio de la Presidencia, Relaciones con las Cortes y Memoria Democrática (2020). Real Decreto 463/2020 de 14 de marzo, por el que se declara el estado de alarma para la gestión de la situación de crisis sanitaria ocasionada por el COVID-19. Bol. Off. Estado.

[4] Álvarez M.A., Hernández M.D.R., Jiménez M., Durán Á. (2014). Lifestyle and meabolic syndrome in college students: Differences by gender. Rev. Psicol. PUCP.

[5] Deshpande S., Basil M.D., Basil D.Z. (2009). Factors Influencing Healthy Eating Habits Among College Students: An Application of the Health Belief Model. Health Mark. Q..

[6] Yaguachi R.A., Reyes M.F., Poveda C.L. (2018). Influence of Lifestyles on the Nutritional Status of University Students. Perspect. Nutr. Hum..

[7] Agüero S.D., Díaz G.B., Velásquez K.F., Zúñiga M.D.R.B., Vega C.E., Noel M.R. (2012). Comparison between the quality of life and nutritional status of nutrition students and those of other university careers at the Santo Thomas University in Chile. Nutr. Hosp..

[8] Gómez J.I., Salazar N. (2010). Hábitos Alimenticios en Estudiantes Universitarios de Ciencias de la Salud de Minatitlán.

[9] Gopalakrishnan S., Ganeshkumar P., Prakash M.V., Amalraj V. (2012). Prevalence of overweight/obesity among the medical students, Malaysia. Med. J. Malays..

[10] Rizo M.M., González N.G., Cortés E. (2014). Calidad de la dieta y estilos de vida en estudiantes de ciencias de la salud. Nutr. Hosp..

[11] Badir A., Tekkas K., Topan S. (2014). Knowladge of cardiovascular disease in Turkish undergraduate nursing students. Eur. J. Cardiovasc. Nurs..

[12] Larisa M., Koksharov A., Krapiva T. (2020). Food safety practices in catering during the coronavirus COVID-19 pandemic. Foods Raw Mater..

[13] Iddir M., Brito A., Dingeo G., Fernandez Del Campo S.S., Samouda H., La Frano M.R., Bohn T. (2020). Strengthening the Immune System and Reducing Inflammation and Oxidative Stress through Diet and Nutrition: Considerations during the COVID-19 Crisis. Nutrients.

[14] Betsch C., Wieler L.H., Habersaat K. (2020). Monitoring behavioural insights related to COVID-19. Lancet.

[15] Brooks S.K., Webster R.K., Smith L.E., Woodland L., Wessely S., Greenberg N., Rubin G.J. (2020). The psychological impact of quarantine and how to reduce it: Rapid review of the evidence. Lancet.

[16] Castañeda-Babarro A., Arbillaga-Etxarri A., Gutiérrez-Santamaría B., Coca A. (2020). Physical activity change during COVID-19 confinement. Int. J. Environ. Res. Public Health.

[17] Sahu A., Naqvi W.M. (2020). Quarantine exercises in the time of Covid-19-a review. J. Evol. Med Dent. Sci..

[18] Smith A.S., Jurek B., Grinevich V., Bowen M.T. (2021). The Oxytocin System in Fear, Stress, Anguish, and Pain. Front. Endocrinol..

[19] Cardi V., Leppanen J., Treasure J. (2015). The effects of negative and positive mood induction on eating behaviour: A metaanalysis of laboratory studies in the healthy population and eating and weight disorders. Neurosci. Biobehav. Rev..

[20] Bennett J., Greene G., Schwartz-Barcott D. (2013). Perceptions of emotional eating behavior. A qualitative study of college students. Appetite.

[21] Lavender J.M., De Young K.P., Wonderlich S.A., Crosby R.D., Engel S.G., Mitchell J.E., Crow S.J., Peterson C.B., Le Grange D. (2013). Daily patterns of anxiety in anorexia nervosa: Associations with eating disorder behaviors in the natural environment. J. Abnorm. Psychol..

[22] Pontes-Torrado Y., García-Villaraco A., Hernández-Galiot A., Goñi -Cambrodón I. (2015). A strategy for weight loss based on healthy dietary habits and control of emotional response to food. Nutr. Hosp..

[23] Taylor S.J., Bogdan R. (1978). Introducción a los Métodos Cualitativos de Investigación (Vol. 1).

[24] Flaudias V., Iceta S., Zerhouni O., Rodgers R.F., Billieux J., Llorca P.M., Boudesseul J., De Chazeron I., Romo L., Maurage P. (2020). COVID-19 pandemic lockdown and problematic eating behaviors in a student population. J. Behav. Addict..

[25] Ruiz-Roso M.B., de Carvalho Padilha P., Mantilla-Escalante D.C., Ulloa N., Brun P., Acevedo-Correa D., Arantes Ferreira Peres W., Martorell M., Aires M.T., de Oliveira Cardoso L. (2020). Covid-19 Confinement and Changes of Adolescent’s Dietary Trends in Italy, Spain, Chile, Colombia and Brazil. Nutrients.

[26] Márquez-Sandoval Y.F., Salazar-Ruiz E.N., Macedo-Ojeda G., Altamirano-Martínez M.B., Bernal-Orozco M.F., Salas-Salvadó J., Vizmanos-Lamotte B. (2014). Diseño y validación de un cuestionario para evaluar el comportamiento alimentario en estudiantes mexicanos del área de la salud. Nutr. Hosp..

[27] Niño Mora V.A. (2021). Hábitos Alimentarios y Estilos de Vida Durante el Confinamiento por COVID-19 en las Familias de los Estudiantes de la Institución Educativa Magdalena. Sogamoso, Boyacá. Bachelor’s Thesis.

[28] Moreno C., Lomelí D., Valencia D. (2021). Actividad física y hábitos alimenticios en universitarios durante la pandemia por COVID-19. I n Memorias de Extenso.

[29] Pérez-Rodrigo C., Gianzo Citores M., Hervás Bárbara G., Ruiz-Litago F., Casis Sáenz L., Arija V., López-Sobaler A.M., Martínez de Victoria E., Ortega R.M., Partearroyo T. (2021). Patrones de cambio de hábitos alimentarios y actividad física durante el confinamiento en España por la pandemia de COVID-19. Nutrients.

[30] Rodríguez-Pérez C., Molina-Montes E., Verardo V., Artacho R., García-Villanova B., Guerra-Hernández E.J., Ruíz-López M.D. (2020). Changes in dietary behaviours during the COVID-19 outbreak confinement in the Spanish COVIDiet Study. Nutrients.

[31] Cancello R., Soranna D., Zambra G., Zambon A., Invitti C. (2020). Determinants of the Lifestyle Changes during COVID-19 Pandemic in the Residents of Northern Italy. Int. J. Environ. Res. Public Health.

[32] López-Bueno R., Calatayud J., Casaña J., Casajús J.A., Smith L., Tully M.A., Andersen L.L., López-Sánchez G.F. (2020). COVID-19 Confinement and Health Risk Behaviors in Spain. Front. Psychol..

[33] Robertson M., Duffy F., Newman E., Bravo C.P., Ates H.H., Sharpe H. (2021). Exploring changes in body image, eating and exercise during the COVID-19 lockdown: A. UK survey. Appetite.

[34] Pearl R. (2020). Weight Stigma and the “Quarantine-15”. Obesity.

[35] Flint S. (2020). Stigmatizing media portrayal of obesity during the coronavirus (COVID-19) pandemic. Front. Psychol..

[36] Cadena-Duarte L., Cardozo L. (2021). Percepción del autoconcepto físico en estudiantes universitarios en tiempos de confinamiento por COVID-19. Cuad. Psicol. Deporte.

[37] Elmacıoğlu F., Emiroğlu E., Ülker M.T., Kırcali B., Oruç S. (2021). Evaluation of nutritional behaviour related to COVID-19. Public Health Nutr..

[38] Coakley K.E., Le H., Silva S.R., Wilks A. (2021). La ansiedad se asocia con rasgos apetitivos en estudiantes universitarios durante la pandemia de COVID-19. Nutr. J..

[39] Padilla P.R., Celi-Torres D., Moreno-Pajuelo A., Lama-Martínez E., Ávalos-Pérez M., Delgado-López V. (2021). CAP-COVID: Conocimientos, actitudes y prácticas (CAP) entorno a la alimentación durante la pandemia de COVID-19 en las ciudades capital de Ecuador y Perú. Nutr. Clín. Y Diet. Hosp..

